# Sheath properties in active magnetized multi-component plasmas

**DOI:** 10.1038/s41598-021-88894-1

**Published:** 2021-05-05

**Authors:** M. M. Hatami

**Affiliations:** Physics Department of K. N. Toosi University of Technology, 15418-49611 Tehran, Iran

**Keywords:** Plasma physics, Magnetically confined plasmas

## Abstract

Multi-component active plasmas are modeled in the presence of a constant oblique magnetic field by using the hydrodynamics equations. Assuming the electrons and negative ions have Boltzmann distribution and the positive ions have finite temperature, the sheath formation criterion is derived by analyzing the Sagdeev potential. It is found that the Bohm velocity of positive ions depends sensitively on the plasma parameters such as ion-neutral collision frequency, electron impact ionization frequency, positive and negative ion temperatures, initial densities of the charged particles and direction of the applied magnetic field. Also, using our obtained Bohm criterion, the sheath properties of an active magnetized plasma consisting of electrons and positive and negative ion species are investigated numerically and the results are compared with the results of a similar quiescent plasma.

## Introduction

Sheaths always surround a plasma boundary and depend on the plasma and wall conditions. The characteristics of the sheaths are critical to the operation of all devices which depend on plasmas such as light sources to materials processing plasmas, fusion plasmas and space physics^1,2^. The Bohm criterion is one of the most famous results in plasma physics because knowing the speed at which ions leave a plasma is important for determining how a plasma interacts with a material boundary^3,4^.

In 1949 Bohm showed that stationary sheath exists if the entrance velocity of ions into the sheath becomes equal to or greater than the so called Bohm velocity $c_{si}=\sqrt{kT_e/m_i}$ where *k* is the Boltzmann constant, $T_e$ is the electron temperature and $m_i$ is the ion mass^5^. As it is seen, this criterion allows to choose uniquely the ion sound speed as the magnitude of the ion velocity at the entrance of the sheath for a plasma with a single ion species^6^. In recent years, this criterion was generalized to the case of magnetized collisional plasma sheathes with thermal ions^7–11^.

The conditions are somewhat complicated in multi-component plasmas such as dusty plasma, electronegative plasma with two negative components and electropositive plasma with two species of positive ions. Several researchers investigated the Bohm criterion and plasma sheath properties in the multi-component plasmas. For example, Wang et al.^12^ investigated sheath structure in a collisionless electronegative plasma consisting of electrons, cold positive ions and hot negative ions. Also, the sheath criterion for a magnetized collisional electronegative plasma consists of hot electrons, hot negative ions and cold positive ions was investigated by Zou et al.^13^. Furthermore, considering the thermal effect of positive ions, the generalized Bohm criterion for a collisonless two-component electron-ion plasma was formulated by Dubinov and Senilov^14^. In addition, Shaw et al.^15^ investigated the properties of a magnetized multi-component plasma sheath consisting of two species of warm positive ions, negative ions and electrons by using three fluid hydrodynamic model. Moreover, a generalized Bohm criterion in the plasma including hot electrons and multiply charged ions was analytically obtained in Ref.^16^. It should be noted that in the above mentioned works, the ionization effect has been ignored. However, some authors have studied the plasma sheath problem by considering the presence of ionization process. For example, Gyergyek and Kovacic^17^ have studied the plasma-wall transition problem in a magnetized electropositive plasma by considering elastic collisions between ions and electrons, and creation/ annihilation of charged particles. Also, Adhikari et al.^18^ have analyzed the influence of the forces that control the ion dynamics inside a magnetized electropositive plasma sheath under collisionless conditions. Furthermore, a criterion of sheath formation has been obtained by Moulick et al.^19^ for magnetized low pressure electropositive plasmas including the effect of collision and ionization. It is worthwhile to mention that in the generalization of the Bohm criterion for multi-component plasmas, similar to what happens in the single ion species plasmas, some authors have assumed that every species of ion reaching the sheath edge at its own Bohm velocity^7,20,21^. However, this assumption did not prove by experimental results of two-ion species plasmas^9,22–24^. In these experiments, the measured velocity of each ion species was closer to the system sound velocity, $c_{cs}=\left( \sum _i \displaystyle \frac{n_{0i}}{n_{0e}}c_{si}^2\right) ^{1/2}$, than their individual sound velocities $c_{si}$ where $n_{0e}$ and $n_{0i}$ are electron and ion densities at the sheath edge.

In the present paper, we extend the results of the previous works (for example, Refs.^11,18–20,25–30^) to include an oblique external magnetic field, ion-neutral collision and electron impact ionization as well as multi-charged positive and negative ion species and find analytically the sheath formation criterion in a magnetized multi-component plasma with ion source term (active plasma) consisting of multi-charged positive and negative ion species. In order to do this, we use a fluid model of plasma and analyze the Sagdeev potential. Considering $E\times B$ drift at the sheath edge, the ions are assumed to enter obliquely into the sheath region. Finally, using the presented Bohm criterion, the properties of the sheath region of an active magnetized plasma consisting of both positive and negative ion species and electrons are investigated numerically and compared with the results of a source-free plasma (quiescent plasma). It should be mentioned that the present work has important industrial applications for modifying surface properties (for example, dry etching and the cleaning of surfaces by sputtering) to enhance the properties of materials^1^.

This work is organized in four sections including the introduction as the first section. In “Model and basic equations” section, we explain our model and basic equations. In “Results and discussion” section, we calculate the modified Bohm criterion analytically and examine it in some interesting physical conditions and finally a brief conclusion is presented in “Conclusion” section.

## Model and basic equations

In this section, we are going to use a fluid model to study the plasma sheath structure in a magnetized plasma consisting of electrons, multi-charged positive and negative ion species and neutral atoms. The quasineutrality condition at the sheath edge of such a plasma system is

1 $$\begin{aligned} n_{0e}+\sum _jQ_{nj}n_{0nj}=\sum _iQ_{pi}n_{0pi}, \end{aligned}$$

where $Q_{pi}$, $Q_{nj}$, $n_{0pi}$ and $n_{0nj}$ are the charge number and density of the ith positive ion and jth negative ion species, respectively and $n_{0e}$ is the electron density at the sheath edge. For the positive ions, the equation of continuity and motion in the steady states are described as

2 $$\begin{aligned}&\nabla .(n_{pi}{\mathbf {v}}_{i})=\nu _In_e, \end{aligned}$$

3 $$\begin{aligned}&\left( {\mathbf {v}}_{i}.\nabla \right) {\mathbf {v}}_{i}=\frac{eQ_{pi}}{m_{i}}\left( {\mathbf {E}}+{\mathbf {v}}_{i}\times {\mathbf {B}}\right) -\frac{1}{m_in_{pi}}\nabla P_i-\nu _{i0}{\mathbf {v}}_{i}, \end{aligned}$$

where $n_{pi}, {\mathbf {v}}_{i}$, $m_{i}$, $P_i={kT_{pi}n_{pi}}^{\gamma _i}/n_{0pi}^{\gamma _{i-1}}$ and $T_{pi}$ are the density, velocity, mass, partial pressure and temperature of the ith positive ion, respectively, $\nu _I$ is ionization frequency, $\nu _{i0}$ is the effective collision frequency, $\gamma _i=1$ is for isothermal approximation and $\gamma _i=3,2,5/3$ for unidimensional, bidimensional, or tridimensional adiabatic flow, respectively. However, Riemann has shown that the fluid approximation for a plane sheath is established if the ion flow is adiabatic^6^.

We assume that an external constant magnetic field ${\mathbf {B}}$ is applied on the sheath region (in the $x-z$ plane) and makes angle $\theta$ with the *x* direction (${\mathbf {B}}/B=(cos\theta , 0, sin\theta$). The *x* direction is taken as the depth direction from the plasma edge to the wall, and the boundary between the plasma $(x < 0)$ and sheath $(x > 0)$ is the plane of $x=0$. Assuming that the external magnetic field is weak and the low-pressure plasma is not highly electronegative, the Boltzmann relations is satisfied well for distribution of electron density^9^

4 $$\begin{aligned} n_{e}=n_{0e}\exp \left( \frac{e\varphi }{kT_{e}}\right) , \end{aligned}$$

where $\varphi$ is the electrostatic potential and $T_e$ is the electron temperature. In addition, the wall potential is specified by a given value $\varphi _{w}$ which can be the floating potential or a more negative value for which Boltzmann distribution holds, i.e., $\mid \varphi _{w}\mid >3kT_{e}/e$^9^. Therefore, the negative ion density distributions are written as follows^9,31^:

5 $$\begin{aligned} n_{nj}=n_{0nj}\exp \left( \frac{eQ_{nj}\varphi }{kT_{nj}}\right) , \end{aligned}$$

where $T_{nj}$ is the temperature of the jth negative ion species.

The Poisson’s equation for such a plasma consisting of multi-charged positive and negative ion species and electrons is written as follows:

6 $$\begin{aligned} \nabla ^{2}\varphi =\frac{e}{\varepsilon _{0}}\left[ n_{e}+\sum _jQ_{nj}n_{nj}-\sum _iQ_{pi}n_{pi}\right] , \end{aligned}$$

where $\varepsilon _{0}$ is the electric permittivity of free space.

Assuming the wall is infinitely long in y and z direction, the quantities change only in *x* direction normal to the wall, i.e., $\nabla \longrightarrow {\hat{x}} \partial /\partial x$. Therefore, the basic equations of the fluid model, i.e., Eqs. (2), (3) and (6) take the following forms:

7 $$\begin{aligned}&\frac{\partial }{\partial x}\left( {n}_{pi}v_{ix}\right) =\nu _In_e, \end{aligned}$$

8 $$\begin{aligned}&v_{ix}\displaystyle \frac{\partial v_{ix}}{\partial x}=\frac{e{Q}_{pi}}{m_i}\left( -\displaystyle \frac{\partial \varphi }{\partial x}+Bv_{iy}sin\theta \right) -\displaystyle \frac{{kT}_{pi}}{m_i{n}_{pi}}\frac{\partial }{\partial x}\left( \displaystyle \frac{{n}_{pi}^{\gamma _i}}{{n}_{0pi}^{\gamma _{i-1}}}\right) -\nu _{i0}v_{ix}, \end{aligned}$$

9 $$\begin{aligned}&v_{ix}\frac{\partial v_{iy}}{\partial x}=\displaystyle \frac{e{Q}_{pi}}{m_i}B\left( v_{iz}cos\theta -v_{ix}sin\theta \right) -\nu _{i0}v_{iy}, \end{aligned}$$

10 $$\begin{aligned}&v_{ix}\frac{\partial v_{iz}}{\partial x}=\displaystyle \frac{-e{Q}_{pi}}{m_i}Bv_{iy}cos\theta -\nu _{i0}v_{iz}, \end{aligned}$$

11 $$\begin{aligned}&\frac{\partial ^2\varphi }{\partial x^2}=\frac{e}{\varepsilon _{0}}\left( n_{e}+\sum _jQ_{nj}{n}_{nj}-\sum _i{Q}_{pi}{n}_{pi}\right) . \end{aligned}$$

The following normalizations have been introduced in order to express the equations (4), (5) and (7)–(11), in terms of the fundamental plasma quantities:

$$\begin{aligned}&{\mathbf {u}}_i={\mathbf {v}}_i/c_{si},\quad {N}_{pi}={n}_{pi}/n_{0e},\quad {N}_{nj}={n}_{nj}/n_{0e},\quad N_e=n_e/n_{0e},\quad \tau _{pi}={T}_{pi}/T_e,\quad \tau _{nj}={T}_{nj}/T_e, \\&X=x/\lambda _{De},\quad \rho =\omega _{ci}/\omega _{pi},\quad \alpha _i=\nu _{i0}/\omega _{pi}, \quad \eta =e\varphi /kT_e,\quad {\delta }_{pi}={n}_{0pi}/n_{0e},\quad {\delta }_{nj}={n}_{0nj}/n_{0e},\quad \nu _{pi}=\nu _I/\omega _{pi}, \end{aligned}$$

where $\lambda _{De}=(\varepsilon _{0}kT_{e}/n_{0e}e^{2})^{1/2}$ is the electron Debye length, and $c_{si}=(kT_e/m_i)^{1/2}$, $\omega _{ci}=eB/m_i$ and $\omega _{pi}=(n_{0pi}e^2/\varepsilon _0 m_i)^{1/2}$ are the cold-ion-acoustic speed, gyrofrequency and plasma frequency for the ith positive ion, respectively.

With these dimensionless quantities the normalized set of the model equations can be written as follows:

12 $$\begin{aligned}&\displaystyle \frac{\partial {N}_{pi}}{\partial X}=\displaystyle \frac{{Q}_{pi}{N}_{pi}{\delta }_{pi}^{\gamma _i-1}}{{u_{ix}^{2}-\gamma _{i}{\tau }_{pi}{N}_{pi}^{\gamma _i-1}}}\left[ \displaystyle \frac{\partial \eta }{\partial X}-\rho {\delta }_{pi}^{1/2} u_{iy}\sin \theta +\displaystyle \frac{u_{ix}{\delta }_{pi}^{1/2}}{{Q}_{pi}}\left( \alpha _{i}+\displaystyle \nu _{pi}\displaystyle \frac{N_e}{{N}_{pi}}\right) \right] , \end{aligned}$$

13 $$\begin{aligned}&\frac{\partial u_{ix}}{\partial X}=-\displaystyle \frac{{Q}_{pi}u_{ix}{\delta }_{pi}^{\gamma _i-1}}{{u_{ix}^{2}-\gamma _{i}{\tau }_{pi}{N}_{pi}^{\gamma _i-1}}}\left[ \displaystyle \frac{\partial \eta }{\partial X}-\rho {\delta }_{pi}^{1/2} u_{iy}\sin \theta +\displaystyle \frac{u_{ix}{\delta }_{pi}^{1/2}}{{Q}_{pi}}\left( \alpha _{i}+\gamma _i\nu _{pi}\displaystyle \frac{N_e}{{N}_{pi}}\displaystyle \frac{{\tau _{pi}}}{u_{ix}^2}\left( \displaystyle \frac{{N}_{pi}}{{\delta }_{pi}}\right) ^{\gamma _i-1}\right) \right] , \end{aligned}$$

14 $$\begin{aligned}&\frac{\partial u_{iy}}{\partial X}={Q}_{pi}\rho {\delta }_{pi}^{1/2}\left( \displaystyle \frac{u_{iz}cos\theta -u_{ix}sin\theta }{u_{ix}}\right) -\alpha _{i}{\delta }_{pi}^{1/2}\displaystyle \frac{u_{iy}}{u_{ix}}, \end{aligned}$$

15 $$\begin{aligned}&\frac{\partial u_{iz}}{\partial X}=-{Q}_{pi}\rho {\delta }_{pi}^{1/2}\left( \displaystyle \frac{u_{iy}}{u_{ix}}\right) cos\theta -\alpha _{i}{\delta }_{pi}^{1/2}\displaystyle \frac{u_{iz}}{u_{ix}}, \end{aligned}$$

16 $$\begin{aligned}&\frac{\partial ^2\eta }{\partial X^2}=\left( N_{e}+\sum _j{Q}_{nj}{N}_{nj}-\sum _i{Q}_{pi}{N}_{pi}\right) , \end{aligned}$$

where $$N_e=\exp (\eta )$$ and $${N}_{nj}={\delta }_{nj}\exp ({Q}_{nj}\eta /{\tau }_{nj})$$.

## Results and discussion

In this section, the sheath formation criterion for a magnetized and collisional plasma consisting of multi-charged positive and negative ion species, electrons and neutral atoms is derived. This is one of the most important boundary condition to solve Eqs. (12)–(16).

Integrating Eq. (16) once, one obtains

17 $$\begin{aligned} \left( \frac{\partial \eta }{\partial X}\right) ^2=\left( \frac{\partial \eta }{\partial X}\right) ^2_{X=0}-2S(\eta ,u_{0ix}), \end{aligned}$$

where

18 $$\begin{aligned} S(\eta ,u_{0ix})=\int _0^{\eta }\left( \sum _i{Q}_{pi}{N}_{pi}-N_{e}-\sum _j{Q}_{nj}{N}_{nj}\right) d\eta , \end{aligned}$$

is a quasipotential called the Sagdeev potential. It is obvious that if Eq. (17) has a real number solution, the right hand side of Eq. (17) should be positive. With $S(0,u_{0ix})=0$ and $\partial S(0,u_{0ix})/\partial \eta =0$, the right hand side of Eq. (17) must satisfy the following relation:

19 $$\begin{aligned} \frac{\partial ^2 S(0,u_{0ix})}{\partial \eta ^2}=\left( \displaystyle \sum _i {Q}_{pi}\frac{\partial {N}_{pi} }{\partial \eta }-\displaystyle \frac{\partial N_e}{\partial \eta }-\sum _j{Q}_{nj}\displaystyle \frac{\partial {N}_{nj} }{\partial \eta } \right) _{\eta =0}<0. \end{aligned}$$

From Eq. (12) and definition of the normalized density of electrons and negative ions, we have

20 $$\begin{aligned}&\left( \displaystyle \frac{\partial N_e}{\partial \eta }\right) _{\eta =0}=1, \end{aligned}$$

21 $$\begin{aligned}&\left( \sum _j{Q}_{nj}\displaystyle \frac{\partial {N}_{nj} }{\partial \eta } \right) _{\eta =0}=\displaystyle \frac{{Q}_{nj}^2{\delta }_{nj}}{{\tau }_{nj}}, \end{aligned}$$

and

22 $$\begin{aligned} \left( \displaystyle \sum _i {Q}_{pi}\frac{\partial {N}_{pi}}{\partial \eta }\right) _{\eta =0}=\sum _i \left( \displaystyle \frac{{Q}_{pi}^2{\delta }_{pi}}{u_{0ix}^2-\gamma _i {\tau }_{pi}}\right) \left( 1+\displaystyle \frac{{\delta }_{pi}^{1/2}\left[ \displaystyle \frac{u_{0ix}}{{Q}_{pi}}\left( \alpha _{i}+\frac{\nu _{pi}}{{\delta }_{pi}}\right) -\rho u_{0iy}sin\theta \right] }{(\displaystyle \frac{\partial \eta }{\partial X})_{\eta =0}}\right) . \end{aligned}$$

Taking into account ${\mathbf {E}}\times {\mathbf {B}}$ drift velocity at the sheath edge, we have $u_{0iy}=-E_0\sin \theta /\rho$ where $E_0=-\left( \partial \eta /\partial X \right) _{\eta =0}$. Therefore, from Eqs. (19)–(22), one can find the modified Bohm criterion in an active multi-component plasmas including effects of the magnetic field and collision as follows:

23 $$\begin{aligned} \sum _i \left( \displaystyle \frac{{Q}_{pi}^2{\delta }_{pi}}{u_{0ix}^2-\gamma _i {\tau }_{pi}}\right) \left( cos^2\theta -\displaystyle \frac{{\delta }_{pi}^{1/2}\left[ \displaystyle \frac{u_{0ix}}{{Q}_{pi}}\left( \alpha _{i}+\frac{\nu _{pi}}{{\delta }_{pi}}\right) \right] }{E_0}\right) \le 1+\sum _j\left( \displaystyle \frac{{Q}_{nj}^2{\delta }_{nj}}{{\tau }_{nj}}\right) , \end{aligned}$$

where

24 $$\begin{aligned} \sum _j{Q}_{j}{\delta }_{nj}=\sum _i{Q}_{pi}{\delta }_{pi}-1. \end{aligned}$$

Now we are going to investigate the validity of our modified Bohm criterion by reducing it to some special cases studied previously by authors:

1. Unmagnetized quiescent plasma consisting of singly-charged warm negative and positive ions: Assuming $\theta =0$, $\alpha _1=0$, $\nu _{p1}=0$, $\delta _{p1}=1+ \delta _{n1}$, $Q_{n1}=1$ and $Q_{p1}=1$ in relation (23), we have:
  25 $$\begin{aligned} u_{01x}\ge \left( \displaystyle \frac{\tau _{n1}\left( 1+\delta _{n1}\right) }{\tau _{n1}+\delta _{n1}}+\gamma _1\tau _{p1}\right) ^{1/2}, \end{aligned}$$
  which is the same results of Braithwaite and Allen^32^.
2. Unmagnetized quiescent plasma consisting of multi-charged negative and warm positive ions: Assuming $\theta =0$, $\alpha _1=0$ and $\nu _{p1}=0$ in (23), the sheath formation criterion and the quasineutrality condition derive as follows:
  26 $$\begin{aligned} \sum _i \displaystyle \frac{Q_{pi}^2\delta _{pi}}{u_{0ix}^2-\gamma _i {\tau _{pi}}}\le 1+\sum _j\displaystyle \frac{Q_{nj}^2{\delta _{ni}}}{{\tau _{nj}}}, \end{aligned}$$
  and
  27 $$\begin{aligned} \sum _jQ_{nj}{\delta _{nj}}+1=\sum _iQ_{pi}{\delta _{pi}}, \end{aligned}$$
  which is the same as the Bohm criterion derived in Ref.^16^.
3. Unmagnetized active plasma with electrons and cold positive ions: Ignoring the effect of magnetic field ($\theta =0$), thermal motion of positive ions ($\tau _{pi}=0$) and presence of negative ions ($\delta _{n1}=0$) in relations (23) and (24), we obtain
  28 $$\begin{aligned} u_{01x}\ge -\frac{\alpha _1+\nu _{p1}}{2E_0}+\left[ 1+\left( \frac{\alpha _1+\nu _{p1}}{2E_0}\right) ^2\right] ^{1/2}, \end{aligned}$$
  which is the same results of Ref.^8^.
4. Unmagnetized active plasma with singly-charged warm positive ions: Assuming $\theta =0$, $\delta _{n1}=0$ and $Q_{p1}=1$ in relations (23) and (24), we have
  29 $$\begin{aligned} u_{01x}\ge -\frac{\alpha _1+\nu _{p1}}{2E_0}+\left[ 1+{\tau }_{p1}+\left( \frac{\alpha _1+\nu _{p1}}{2E_0}\right) ^2\right] ^{1/2}. \end{aligned}$$
5. Unmagnetized active plasma with singly-charged negative and warm positive ion: Considering $\theta =0$, $Q_{n1}=1$ and $Q_{p1}=1$ in (23) and (24), we have
  30 $$\begin{aligned} u_{01x}\ge -\frac{{\delta }_{p1}^{3/2} }{2E_0}\frac{(\alpha _1+\frac{\nu _{p1}}{{\delta }_{p1}})}{(1+\frac{{\delta }_{n1}}{{\tau _{n1}}})}+ \left[ \left( \frac{{\delta }_{p1}^{3/2} }{2E_0}\frac{(\alpha _1+\frac{\nu _{p1}}{{\delta }_{p1}})}{(1+\frac{{\delta }_{n1}}{{\tau _{n1}}})}\right) ^2+\frac{{\delta }_{p1}+\gamma _1{\tau _{p1}}(1+\frac{{\delta }_{n1}}{{\tau _{n1}}})}{1+\frac{{\delta _{n1}}}{{\tau _{n1}}}}\right] ^{1/2}. \end{aligned}$$
6. Magnetized quiescent plasma consisting of singly-charged negative and cold positive ions: Assuming $\nu _{p1}=0$, $\alpha _1=0$, $\tau _{p1}=0$, $Q_{p1}=1$ and $Q_{n1}=1$ in inequality (23), we have
  31 $$\begin{aligned} u_{01x}\ge \left( \displaystyle \frac{\delta _{p1}\left( 1-\displaystyle \delta _{p1}^{1/2}sin^2\theta \right) }{1+\displaystyle \frac{\delta _{p1}-1}{\tau _{n1}}}\right) ^{1/2}, \end{aligned}$$
  which is the sheath criterion reported by Zou et al for $\gamma _1=1$^13^.
7. Magnetized quiescent plasma consisting of singly-charged warm positive ions: By considering $\nu _{p1}=0$, $\delta _{n1}=0$, $Q_{p1}=1$, $\alpha _1=0$ and $\delta _{p1}=1$ in relation (23), the sheath criterion can be written as follows:
  32 $$\begin{aligned} u_{01x}\ge \left( \tau _{p1}+cos^2\theta \right) ^{1/2}. \end{aligned}$$
  Relation (32) is the same result of Liu et al.^11^ for isothermal ion flow.
8. Magnetized collisional active plasma with multi-charged warm positive ions: Assuming $\delta _{n1}=0$, relations (23) and (24) reduce to the following form:
  33 $$\begin{aligned}&u_{01x}\ge -\frac{Q_{p1}{\delta }_{p1}^{3/2} }{2E_0}\frac{(\alpha _1+\frac{\nu _{p1}}{{\delta }_{p1}})}{(1+\frac{{\delta }_{n1}}{{\tau _{n1}}})} \nonumber \\&\quad + \left[ \left( \frac{Q_{p1}{\delta }_{p1}^{3/2} }{2E_0}\frac{(\alpha _1+\frac{\nu _{p1}}{{\delta }_{p1}})}{(1+\frac{{\delta }_{n1}}{{\tau _{n1}}})}\right) ^2+\frac{Q_{p1}^2{\delta }_{p1}cos^2\theta +\gamma _1{\tau _{p1}}(1+\frac{{\delta }_{n1}}{{\tau _{n1}}})}{1+\frac{{\delta _{n1}}}{{\tau _{n1}}}}\right] ^{1/2}. \end{aligned}$$
9. Magnetized collisional active plasma consisting of warm multi-charged positive and negative ions, and electrons: In this case, from relations (23) and (24), we have
  34 $$\begin{aligned}&u_{01x}\ge -\frac{Q_{p1}{\delta }_{p1}^{3/2} }{2E_0}\frac{(\alpha _1+\frac{\nu _{p1}}{{\delta }_{p1}})}{(1+Q_{n1}^2\frac{{\delta }_{n1}}{{\tau _{n1}}})} \nonumber \\&\quad + \left[ \left( \frac{Q_{p1}{\delta }_{p1}^{3/2} }{2E_0}\frac{(\alpha _1+\frac{\nu _{p1}}{{\delta }_{p1}})}{(1+Q_{n1}^2\frac{{\delta }_{n1}}{{\tau _{n1}}})}\right) ^2+\frac{Q_{p1}^2{\delta }_{p1}cos^2\theta +\gamma _1{\tau _{p1}}(1+Q_{n1}^2\frac{{\delta }_{n1}}{{\tau _{n1}}})}{1+Q_{n1}^2\frac{{\delta _{n1}}}{{\tau _{n1}}}}\right] ^{1/2}. \end{aligned}$$

Now, we are going to use the generalized Bohm criterion (Eq. 23) to investigate the sheath properties in an active plasma. In order to do this, considering an active plasma ($\nu _I\ne 0$) consisting of electron and positive and negative ions with $T_e=1 eV$, $n_{0p1}=10^{9} cm^{-3}$, $\eta (X=0)=0$ and $E_0=0.01$, the effects of collision and ionization frequencies as well as the presence of the magnetic field on the characteristics of the sheath region are studied numerically by solving Eqs. (12)–(16) using a fourth-order Runge-Kutta method. Also, to better understand the effects of these parameters on the sheath properties, the results of the present work are compared with their counterparts in a collisionless, unmagnetized quiescent plasma $(\nu _I=0)$.

**Figure 1:**
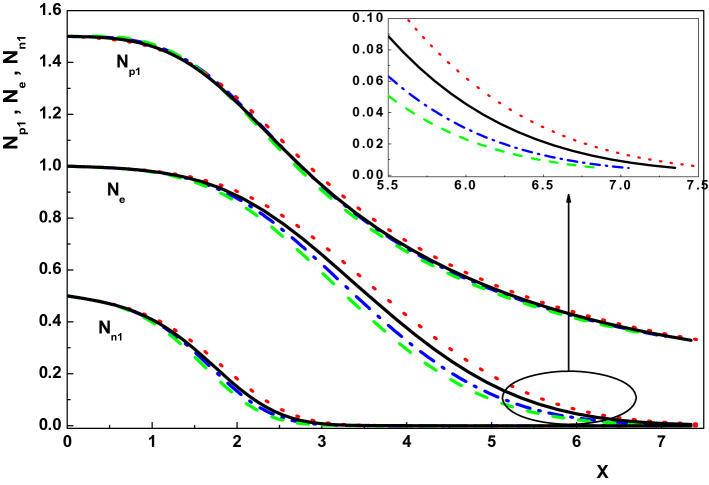
Variation of the normalized density distribution of the charged particles toward the wall for $\nu _{p1}=0, \rho =0, u_{01x}=0.774$ (dotted-dashed line), $\nu _{p1}=0, \rho =0.01, u_{01x}=0.72$ (dotted line), $\nu _{p1}=0.005, \rho =0, u_{01x}=0.774$ (dashed line) and $\nu _{p1}=0.005, \rho =0.01, u_{01x}=0.72$ (solid line).

**Figure 2:**
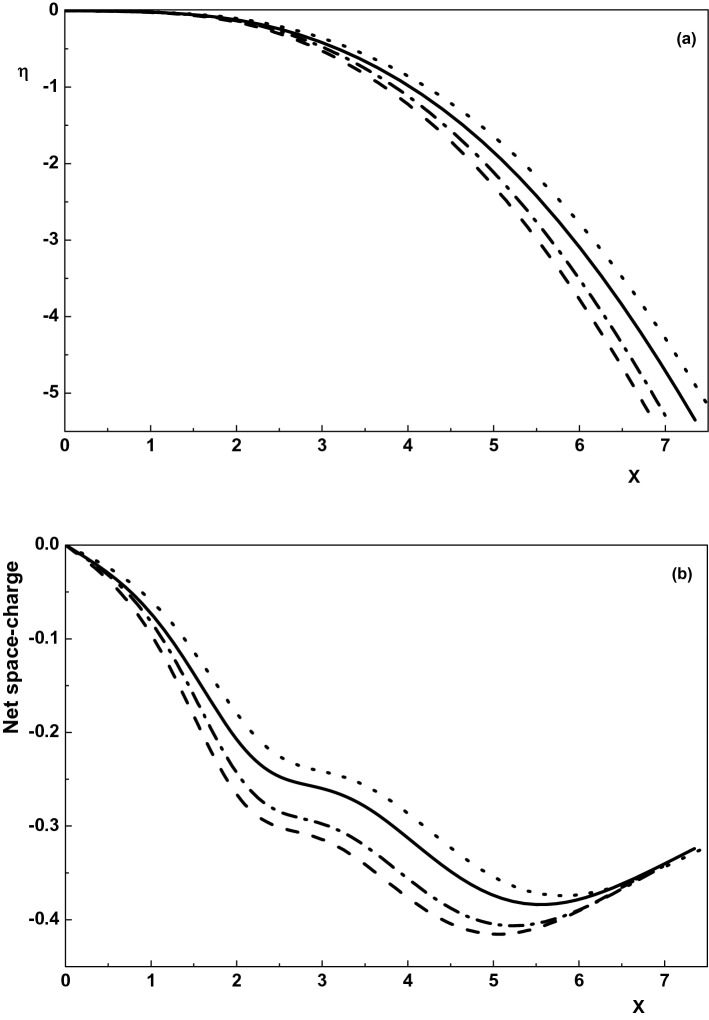
Variation of the normalized (**a**) electric potential and (**b**) net space-charge toward the wall for $\nu _{p1}=0, \rho =0$ (dotted-dashed line), $\nu _{p1}=0, \rho =0.01$ (dotted line),$\nu _{p1}=0.005, \rho =0$ (dashed line) and $\nu _{p1}=0.005, \rho =0.01$ (solid line). Other parameters are the same with Fig. 1.

Figure 1 shows the effect of the magnetic field on the density distribution of the charged particles (electron, positive ion and negative ion) in an active and quiescent plasma for $\gamma _1=3$, $\alpha =0.01$, ${\tau }_{p1}=0.1$, ${\delta }_{n1}=0.5$, ${\tau }_{n1}=0.1$, $\theta =30^\circ$ and different values of magnetic field (via $\rho$), respectively^6,8,28,33–36^. This figure indicates that density distribution of the charged particles and also the sheath width in both active and quiescent plasma increase in the presence of a constant magnetic field which is in agreement with the result of Ref.^37^. The physical reason is the gyral effects of the magnetic force on the positive ions in the sheath. In fact, magnetic force can not only speed up but also slow down the ion flow in the *x*-axis direction. On the other hand, the positive ion density distribution has effects on electron and negative ions by the Poisson’s equation. Therefore, the density distributions of the three kinds of charged particles are higher in the presence of magnetic field. In addition, it is seen that the negative ion density distribution falls down much faster than that for the positive ion and electron in both kind of plasmas ($\nu _I=0$,  $\nu _I\ne 0$). Moreover, comparing the curves in Fig. 1 shows that density of species ($N_{p1}$, $N_{n1}$ and $N_e$) in an active plasma are smaller than those in a quiescent plasma regardless of the presence of the magnetic field.

Effect of the magnetic field on the sheath potential and net space-charge of an active plasma is depicted in Fig. 2a,b for $\gamma _1=1$, $\alpha =0.01$, ${\tau }_{p1}=0.1$, ${\delta }_{n1}=0.5$ and ${\tau }_{n1}=0.1$. It is shown that either in an active plasma or in a quiescent plasma the electrostatic potential (net space-charge) in the sheath region increases by increasing $\rho$. Also, comparing the curves in Fig. 2 shows that either in the presence or absence of the magnetic field the quiescent plasma has a greater net space-charge and potential in the sheath region. Therefore, it can be concluded that the presence of the magnetic field in both active and quiescent collisional plasma with warm positive ions and Maxwellian electrons and negative ions causes the sheath properties (the sheath width, potential, net space-charge and density of species) to increase.

**Figure 3:**
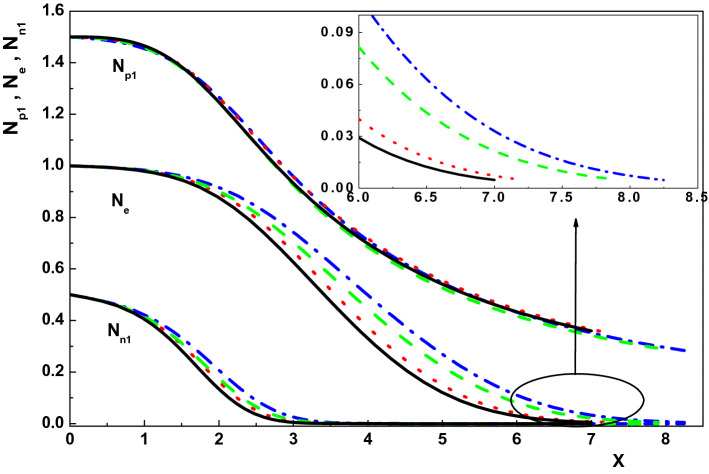
Variation of the normalized density distribution of the charged particles toward the wall for $\nu _{p1}=0, u_{01x}=0.622, \tau _{p1}=0$ (dotted-dashed line), $\nu _{p1}=0, u_{01x}=0.804, \tau _{p1}=0.2$ (dotted line), $\nu _{p1}=0.005, u_{01x}=0.622, \tau _{p1}=0$ (dashed line) and $\nu _{p1}=0.005, u_{01x}=0.804, \tau _{p1}=0.2$ (solid line).

**Figure 4:**
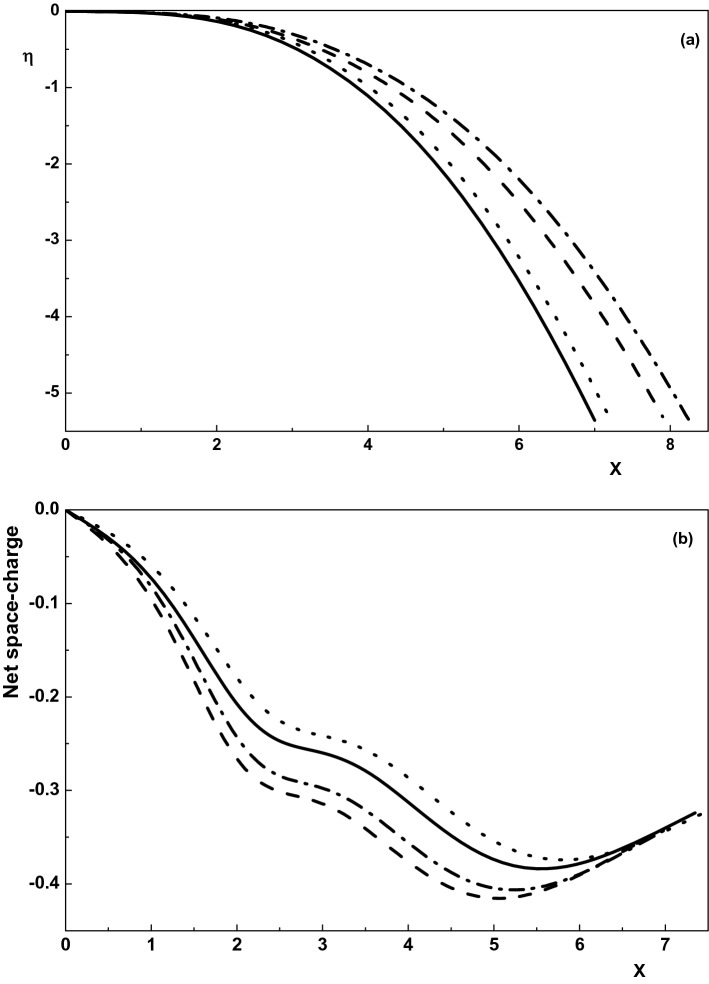
Variation of the normalized (**a**) electric potential and (**b**) net space-charge toward the wall for $\nu _{p1}=0, \tau _{p1}=0$ (dotted-dashed line), $\nu _{p1}=0, \tau _{p1}=0.2$ (dotted line), $\nu _{p1}=0.005, \tau _{p1}=0$ (dashed line) and $\nu _{p1}=0.005, \tau _{p1}=0.2$ (solid line). Other parameters are the same with Fig. 3.

To see the effect of the positive ion temperatures on the sheath properties of an active magnetized multi-component plasma consisting of electrons and positive and negative ions, we plot Figs. 3 and 4 for $\gamma _1=1$, $\alpha =0.01$, $\rho =0.01$, $\theta =30^\circ$, ${\delta }_{n1}=0.5$, ${\tau }_{n1}=0.1$ and different values of ${\tau }_{p1}$. As it is seen from these figures, density of species, net space-charge and the electrostatic potential of the sheath decrease by increasing the temperature of the positive ions in an active magnetized plasma. Also, it can be concluded from Figs. 3 and 4 that the sheath thickness is a decreasing function of the positive ion temperature^38,39^. The reason behind decrease in the sheath width is that by increasing the positive ion temperature, the gradient of the ion pressure increases and so the ions hit the wall more quickly due to the higher velocity entering sheath and the larger ion pressure. This effect, of course, causes decrease in the sheath width by the increase in $\tau _{p1}$. Moreover, similar to Fig. 1, density distribution of the negative ions goes down much faster than the other charged particles in the sheath. Further, comparing the curves of density of species, the electrostatic potential and the net space-charge in an active and quiescent plasma at a fixed ${\tau }_{p1}$ shows that these properties are smaller in an active plasma. As a result, it can be found that the warmness of positive ion species either in an active or quiescent plasma leads to a decrease in the sheath characteristics.

**Figure 5:**
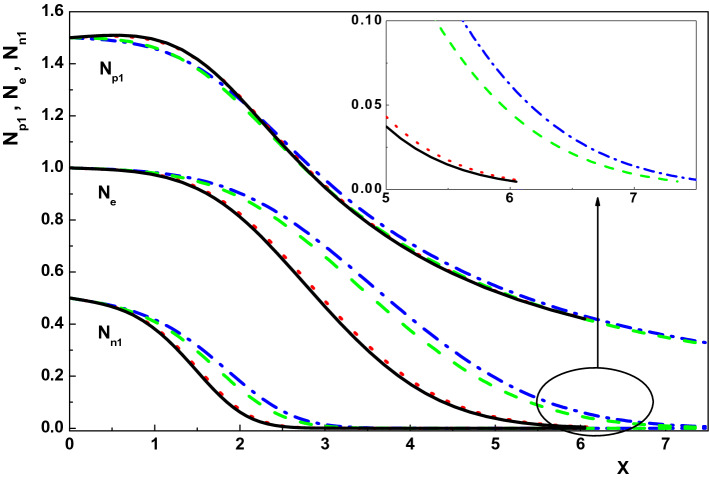
Variation of the normalized density distribution of the charged particles toward the wall for $\nu _{p1}=0, u_{01x}=0.720, \alpha =0.01$ (dotted line), $\nu _{p1}=0, u_{01x}=0.933, \alpha =0.02$ (dotted-dashed line), $\nu _{p1}=0.005, u_{01x}=0.721, \alpha =0.01$ (solid line) and $\nu _{p1}=0.005, u_{01x}=0.935, \alpha =0.02$ (dashed line).

**Figure 6:**
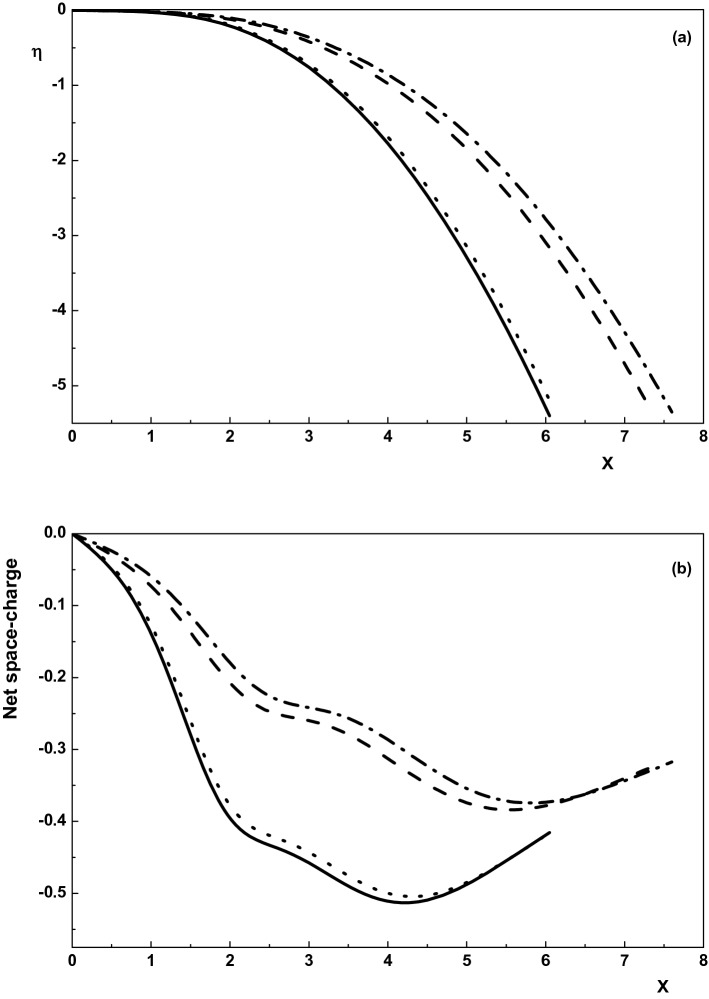
Variation of the normalized (**a**) electric potential and (**b**) net space-charge toward the wall for $\nu _{p1}=0, u_{01x}=0.720, \alpha =0.01$ (dotted line), $\nu _{p1}=0, u_{01x}=0.933, \alpha =0.02$ (dotted-dashed line), $\nu _{p1}=0.005, u_{01x}=0.721, \alpha =0.01$ (solid line) and $\nu _{p1}=0.005, u_{01x}=0.935, \alpha =0.02$ (dashed line).

At the end of this section, we are going to investigate the effect of the collision frequency $\alpha$ on the sheath properties of an active multi-component plasma with finite temperature positive ions in the presence of an oblique magnetic field. In order to do this, we plot Figs. 5 and 6 for ${\tau }_{p1}=0.1$ and different values of $\alpha$. The other plasma parameters are the same with Fig. 3. Figures 5 and 6 indicate that increasing the collision frequency leads to a increase in the charged particle densities, the potential and also the net space-charge in both active and quiescent plasmas. Moreover, similar to Refs.^37,39,40^, the sheath thickness increases by increasing the collision frequency.

## Conclusion

Hydrodynamics equations were used to investigate the plasma sheath criterion and properties in a multi-component active plasma consisting of positive and negative ion species and electrons in the presence of an external oblique magnetic field. It was assumed that the positive ions have finite temperatures and the electrons and negative ions obey the Boltzmann density distribution with different temperatures. Also, considering the $E\times B$ drift at the sheath edge, the positive ions enter obliquely into the sheath region. Using Sagdeev potential to drive the Bohm criterion, the minimum allowable velocity of positive ions at the sheath edge was determined. It was found that this velocity sensitively depends on the plasma parameters such as positive and negative ion-to-electron temperature ratios, direction of the magnetic field, charge number of ion species, ionization and collision frequencies and initial density of the charged particles at the sheath edge. In continuation, properties of the sheath region of an active magnetized plasma consisting of electrons and positive and negative ions were investigated numerically by using the presented generalized Bohm criterion in this work. Our results show that the potential, the net space-charge and density distribution of the charged particles in the sheath, and also the sheath width increase (decrease) as the collision frequency (the temperature of the positive ions) increases in an active magnetized plasma. Further, it was shown that the presence of the magnetic field causes the potential of the sheath, density distribution of species and also the net space-charge in the sheath to increase. Finally, comparing the properties of the sheath region of an active and a quiescent plasma shows that the presence of ionization process causes to decrease of the mentioned sheath properties.
